# Psychosocial risk factors for postpartum depression in Chinese women: a meta-analysis

**DOI:** 10.1186/s12884-021-03657-0

**Published:** 2021-03-02

**Authors:** Weijing Qi, Fuqing Zhao, Yutong Liu, Qing Li, Jie Hu

**Affiliations:** Department of Clinical Humanistic Care and Nursing Research Center, School of Nursing, Hebei Medical University, Dr. 361 East Zhongshan Road, Shijiazhuang, 050017 Hebei Province China

**Keywords:** Postpartum depression, Risk factors, Meta-analysis, Psychosocial

## Abstract

**Background:**

Postpartum depression (PPD) has been identified as a recognized public health problem that may adversely affect mothers, infants, and family units. Recent studies have identified risk factors for PPD in Westerners; however, societal and cultural differences between China and the West could, potentially, lead to differences in risk factors for PPD. No comprehensive study has been conducted to collect all the evidence to provide estimates of psychological and social risk factors in China. Therefore, this study aimed to quantitatively assess all studies meeting the review’s eligibility criteria and identify the psychological and social risk factors for PPD in Chinese women.

**Methods:**

The following databases were used in the literature search from their inception until December 2020: PubMed, Embase, Foreign Medical Literature Retrieval Service (FMRS), China Science and Technology Journal Database (VIP), China National Knowledge Infrastructure (CNKI), and China Biology Medicine disc (CBM). The quality was assessed through Newcastle-Ottawa quality assessment scale. The I^2^statistic was used to quantify heterogeneity. We extracted data for meta-analysis and generated pooled-effect estimates from a fixed-effects model. Pooled estimates from a random-effects model were also generated if significant heterogeneity was present. Funnel plot asymmetry tests were used to check for publication bias. Statistical analysis was conducted using Review Manager version 5.3 software.

**Results:**

From a total of 1175 identified studies, 51 were included in the analysis. Prenatal depression (OR 7.70; 95% CI 6.02–9.83) and prenatal anxiety (OR 7.07; 95% CI 4.12–12.13) were major risk factors for PPD. A poor economic foundation (OR 3.67; 95% CI 3.07–4.37) and a poor relationship between husband and wife (OR 3.56; 95% CI 2.95–4.28) were moderate risk factors. Minor risk factors included a poor relationship between mother-in-law and daughter-in-law (OR 2.89; 95% CI 2.12–3.95), a lack of social support (OR 2.57; 95% CI 2.32–2.85), unplanned pregnancy (OR 2.55; 95% CI 2.08–3.14), and poor living conditions (OR 2.44; 95% CI 1.92–3.10), mother-in-law as the caregiver (1.95; 95% CI 1.54–2.48) .

**Conclusions:**

This study demonstrated a number of psychological and social risk factors for PPD in Chinese women. The major and moderate risk factors are prenatal depression, prenatal anxiety, a poor economic foundation, and a poor relationship between husband and wife. These findings have potential implications for informing preventive efforts and modifying screening to target at-risk populations.

## Background

Postpartum depression (PPD) is the most common type of nonpsychotic psychiatric syndrome during the perinatal period [1]. The *Diagnostic and Statistical Manual of Mental Disorders Fifth Edition* (DSM-5) defines PPD as a depressive episode with moderate-to-severe symptoms that begins 4 weeks after delivery [2]. The prevalence of PPD varies from 0.5 to 60.8% around the world and from 3.5 to 63.3% in Asian countries, as measured using the Edinburgh Postpartum Depression scale (EPDS) [3]. The prevalence of PPD in China is 27.37% [4], and is increasing yearly [5]. PPD has been identified as a recognized public health problem that not only affects mothers’ health but also causes poor developmental outcomes in children and poor relationships in families [6].

Postpartum depressive symptoms include the inability to sleep, anxiety, sadness, extreme concern and worry about the baby, and even recurrent thoughts of death [7]. Because maternal emotion plays an important role in the development of children, the pathogenesis of PPD merits greater attention. At present, studies involving Chinese women have reported that poor relationships with husbands or mothers-in-law, introverted maternal personality, anxiety or depression during pregnancy, an unsmooth delivery process, poor postpartum sleep quality, dissatisfaction with neonatal sex, and poor health conditions of newborns were risk factors for PPD [8]. Although several risk factors have been identified, the results of some risk factors are still controversial. Of note, these reviews included some case-control studies and cross-sectional studies, which limited the strength and quality of such evidence.

In addition, review [9] on the risk factors for PPD have primarily included studies conducted in Western populations and have overlooked many studies undertaken in the Chinese cultural context. Western and Chinese women differ considerably in terms of genetics, philosophical traditions, cultural practices, ethnicity, religion, and attitudes toward psychological problems [10]. The two different cultural backgrounds lead to differences in the psychosocial risk factors for PPD [11]. Given the deeply rooted influence of the Chinese cultural traditions of Confucianism, Buddhism and Taoism, the thought processes of Chinese females have historically been more traditional and conservative in nature [12]. Some studies have shown that culturally and characteristically Westerners are more extroverted, while characteristically Chinese people are more introverted - introverted women with personality types that are dependent are more prone to experience PPD [13, 14].

In addition, although some Chinese individuals have emigrated to other countries, they have been influenced by traditional Chinese culture for a long time; thus their personality and their family structure are still similar to those of individuals in their motherland. In particular, many Chinese immigrant women continue to observe the Chinese traditional postpartum practices. Thus the aim of this review was to synthesis the evidence from eligible studies to identify the psychosocial risk factors for PPD in Chinese women, including those living in other countries.

## Methods

### Search strategy

A systematic search of the electronic databases PubMed, Embase, Foreign Medial Literature Retrieval Service (FMRS), the China Science and Technology Journal Database (VIP), China National Knowledge Infrastructure (CNKI), and China Biology Medicine disc (CBM) was performed for relevant studies published before June 2019. Chinese search terms included PPD, risk factors, influencing factors, social factors, and psychological factors. English search terms included the following: Postnatal Depression OR Depression, Postnatal OR Post-Partum Depression OR Depression, Post-Partum OR Post Partum Depression OR Postpartum Depression OR Post-Natal Depression OR Depression, Post-Natal OR Post Natal Depression AND Factor, Risk OR Factors, Risk OR Risk Factor OR Population at Risk OR Risk, Population at OR Populations at Risk OR Risk, Populations at AND Chinese OR in China. Additionally, we also performed a manual search of the reference lists of retrieved articles and recent reviews.

### Population (P)

Chinese women who have given birth to at least one child, including those living in countries other than China.

### Exposure (E)

A woman who has experienced childbirth and has been exposed to a risk factor for PPD.

### Outcome (O)

PPD is the most common type of nonpsychotic psychiatric syndrome during the perinatal period, as a depressive episode with moderate-to-severe symptoms that begins 4 weeks after delivery.

### Selection criteria

This systematic review and meta-analysis considered all studies on psychosocial risk factors for PPD in Chinese women, including Chinese women currently living in other countries.

### Exclusion criteria

The title and abstracts of retrieved studies were assessed against the review’s inclusion criteria. Papers that were inaccessible, and those that did not report associated risk factors for PPD were excluded. Studies that were not a case-control study or a cohort study were also excluded. Once more, studies that did not report relative risks (RRs) or odds ratios (ORs) with corresponding 95% confidence intervals (CIs) or the numbers of women with PPD were also excluded from this review.

### Data extraction and quality assessment

Data extraction and quality assessment was done by two reviewers independently. Disputes were discussed between the reviewers until consensus was reached. Data extracted from the studies included the first author, year, study sites, sample size, PPD Group, assessment method, quality score, investigation time, as well as risk factors associated with PPD. Articles that fulfilled the predefined criteria were used as a source of data for the final analysis.

The instrument versions were Chinese. The risk factors were assessed by questionnaire and interview. We used the Newcastle-Ottawa Scale (NOS) to assess the quality of the selected cohort and case-control studies [15]. The NOS was used to score the studies on three criteria: the selection of the study groups; the comparability of the groups; and the ascertainment of outcome or exposure. The total score ranged from 0 to 9, with higher scores representing higher methodological quality and lower risk of bias.

### Statistical analysis

The number of cases of PPD and the number of cases in the control group were converted into the form of odds ratios (ORs) with 95% confidence intervals (CIs), which were used to pool the outcome data. The Cochrane Q test was performed to assess statistical heterogeneity, and the Higgins I^2^ statistic was used to determine the extent of variation between effect estimates (0 to 100%). For outcomes with low heterogeneity, I^2^ < 50% and *p* > 0.1, the fixed-effects model (M-H method) was used for analysis. In addition to I^2^ ≥ 50% or *p* < 0.1, the random-effects model (D-l method) was used [16]. The sensitivity analysis was carried out by changing the model method. Publication bias was evaluated via the visual analysis of funnel plots. In the absence of bias, showing a symmetrical inverted funnel. When drawing a funnel chart, at least 10 studies are needed. The funnel plot may not detect publication bias when the number of studies is small [17]. Statistical analyses were performed using Review Manager 5.3 (Cochrane Collaboration, UK).

## Results

### Literature search

**Fig. 1:**
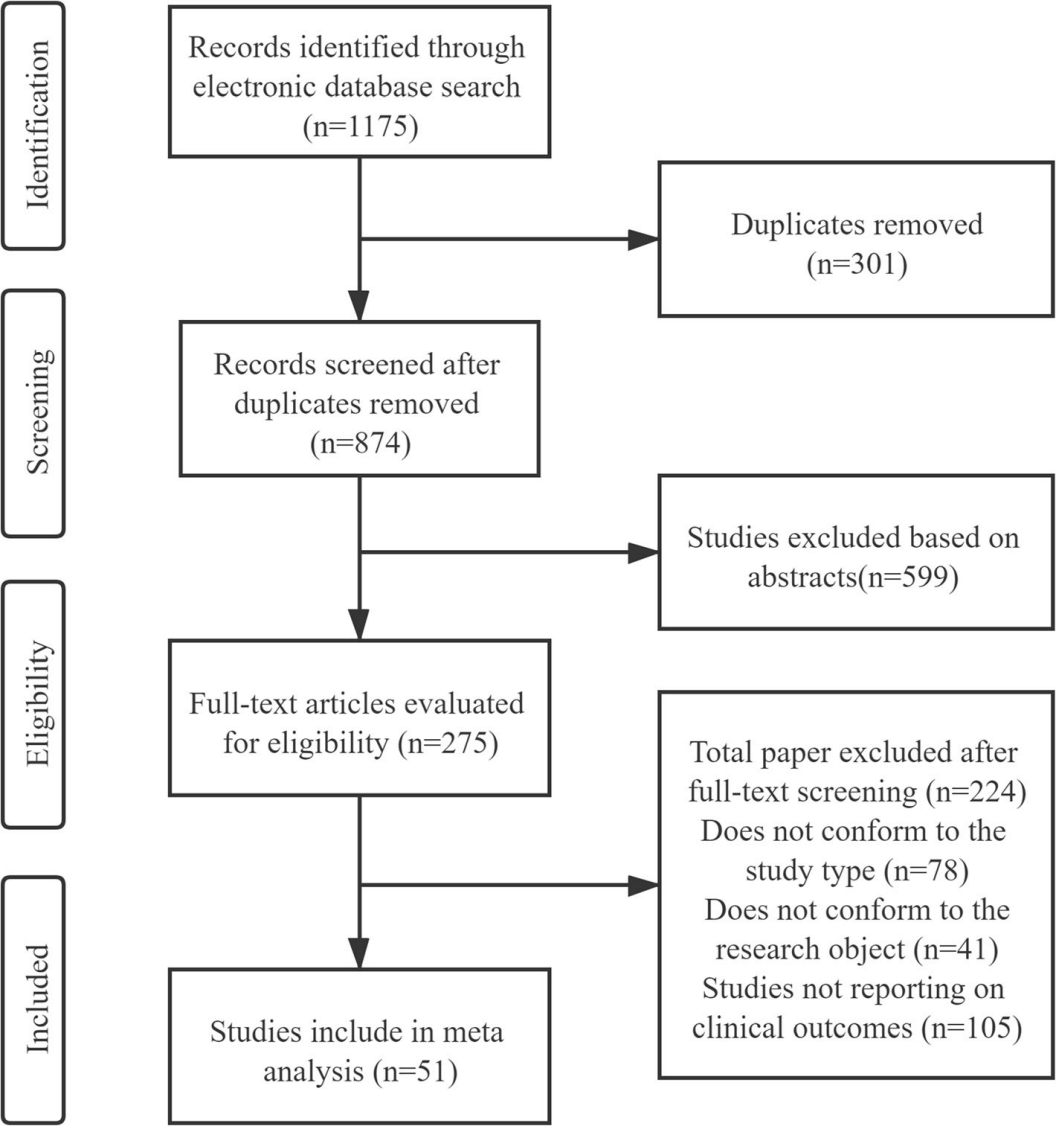
Flowchart steps of the meta analysis

A total of 1175 studies were obtained through six database searches, of which 301 were excluded because of duplicates. Most articles (*n* = 599) were excluded after the title and abstract information were reviewed. Then, 224 articles that did not meet the inclusion criteria were excluded after the full text was reviewed. Finally, the meta-analysis included 51studies, and the study selection process is shown in Fig. 1.

### Study characteristics

A total of 36,705 perinatal women were included across the 12 cohort studies and 39 case-control studies, with 7008 women identified as having PPD. The studies investigated Chinese women in mainland China and Hong Kong, and Canadian immigrants. The EPDS and Self-Rating Depression Scale (SDS) were the most common instruments used to assess PPD. The quality score of the included studies ranged from 5 to 8, and 13 studies were scored 7 or more. The study characteristics and quality evaluation are summarized in Table 1.

**Table 1:** Characteristics of included studies

First author	Year	study sites	Sample size	PPD Group	Assessment method	Quality scores	Investigate time	Risk factors
Lee [18]	2004	Hong Kong	781	122	EPDS	8	first trimester/ 6 weeks postpartum	(1),(5)
Zhao [19]	2018	Shanghai	215	67	EPDS	7	late pregnancy/ week 3-days and 6-weeks after delivery	(1)
Pan [20]	2004	Sichuan	427	33	EPDS	6	third trimester/ 4 ~ 6 weeks postpartum	(1),(3)
Zhao [21]	2018	Sichuan	1440	25	EPDS	6	third trimester/ 8,18 weeks postpartum	(1)
Siu [18]	2012	Hong Kong	805	126	EPDS	7	third trimester/ around 2 months postnatally	(4),(5)
Li [22]	2017	Shanxi	1759	593	EPDS	6	third trimester/ 4 weeks postpartum	(4),(6)
Gu [23]	2004	Shanghai	999	307	HADS	6	Second trimester/ 4 weeks postpartum	(3),(4)
Sun [24]	2015	Yunnan	528	96	EPDS	6	third trimester/ 4 weeks postpartum	(6)
Kang [25]	2015	Jiangsu	3972	468	EPDS> 10	6	first trimester/ 6 weeks postpartum	(6)
Dennis [26]	2017	Canadian immigrants	549	120	EPDS> 9	8	third trimestery/ 4 weeks postpartum	(6)
Cai [27]	2017	Chongqing	371	60	EPDS≥13	7	Second trimester/ 6 weeks postpartum	(6)
Hu [28]	2010	Sichuan	264	146	EPDS> 9	6	Second trimester/ 4 weeks postpartum	(4)
Gu [29]	2017	Xinjiang	824	286	EPDS> 13	6	42 days postpartum	(1),(2)
Zhang [30]	2017	Guangdong	538	49	EPDS> 10	7	42 days postpartum	(1),(2)
Wu [31]	2019	Guangdong	1437	100	EPDS> 10	6	4 weeks postpartum	(1),(2)
Shen [32]	2011	Shanxi	104	52	EPDS> 13	6	4 weeks postpartum	(1)
Yang [33]	2016	Hubei	400	37	EPDS> 13	6	4 ~ 6 weeks postpartum	(1),(3),(8)
Zhang [34]	2001	Tianjin	463	47	EPDS≥13	7	4 weeks postpartum	(4)
Yin [35]	2011	Guangdong	202	37	SDS > 40	5	4 weeks postpartum	(3),(4)
Huang [36]	2012	Hunan	302	56	EPDS	6	6 ~ 7 weeks postpartum	(4)
Song [37]	2012	Hunan	285	69	SDS	5	4 weeks postpartum	(3),(4),(5)
Wang [38]	2013	Beijing	435	27	SCL-90 > 2	6	42 days postpartum	(4)
Lin [39]	2014	Zhejiang	2023	204	EPDS	5	42 days postpartum	(4)
Zhou [40]	2014	Hubei	378	294	EPDS/SDS	5	4 ~ 6 weeks postpartum	(4)
Wang [41]	2014	Guizhou	875	112	EPDS	7	42 days postpartum	(3),(4),(8)
Chen [42]	2017	Zhejiang	380	260	EPDS> 13	7	42 days postpartum	(4),(8)
Jiang [43]	2018	Shandong	185	25	EPDS	5	6 weeks postpartum	(4)
Han [44]	2018	Henan	248	124	EPDS> 10	5	4 weeks postpartum	(4)
He [45]	2019	Zhejiang	398	217	SDS EPDS	5	4 weeks postpartum	(4)
Yu [46]	2010	Shanghai	673	73	EPDS	7	6 weeks postpartum	(3),(7)
Zhang [47]	2012	Hunan	215	67	EPDS> 13	6	30 ~ 42 days postpartum	(7)
Zhang [48]	2014	Guangdong	586	87	EPDS> 13	6	6 weeks postpartum	(7)
Li [49]	2014	Anhui	687	103	EPDS	7	6 weeks postpartum	(3),(7)
Liu [50]	2015	Hunan	232	43	EPDS/HAD	5	42 days postpartum	(7)
Liu [51]	2015	Heilongjiang	576	162	EPDS	6	42 days postpartum	(6),(7)
Chen [52]	2018	Northwest China	640	84	EPDS> 13	6	6 weeks postpartum	(7),(9)
Wang [53]	2013	Shandong	917	168	EPDS/SDS	5	6 weeks postpartum	(3)
Li [54]	2019	Shanxi	170	85	SDS	5	6 weeks postpartum	(3),(6)
Guan [55]	2012	Inner Mongolia	246	92	EPDS> 9	6	30 ~ 42 days postpartum	(9)
Zhang [56]	2011	Hubei	479	167	BDI > =5	6	7 ~ 30 days postpartum	(9)
Deng [57]	2014	Guangdong	2021	158	CES-D > 20	6	42 days postpartum	(5)
Deng [4]	2014	Guangdong	1823	499	EPDS> 13	7	4 weeks postpartum	(5)
Han [58]	2015	Beijing	203	189	EPDS> 13 HAD> 9	6	42 days postpartum	(5),(8)
Liu [59]	2017	Guangdong	418	93	EPDS> 9.5	5	4 ~ 6 weeks postpartum	(5)
Zhou [60]	2019	Jiangsu	849	142	EPDS> 10	6	2 ~ 6 weeks postpartum	(5)
Liu [61]	2015	Hubei	1427	198	EPDS≥9	5	42 days postpartum	(6),(8)
Xie [62]	2018	Hubei	534	103	EPDS	6	6 weeks postpartum	(6)
Pan [63]	2015	Zhejiang	745	93	EPDS	5	42 days postpartum	(2)
Yang [64]	2020	Jiangxi	371	94	EPDS	6	42 days postpartum, third trimester,	(3),(4),(9)
Huang [65]	2020	Jiangsu	784	59	EPDS	6	42 days postpartum	(3)
Qing [66]	2020	Guangdong	522	90	EPDS	7	2nd to 4th postnatal months	(9)

Risk factors: (1) Prenatal depression, (2) prenatal anxiety, (3) poor economic foundation, (4) poor relationship between husband and wife, (5) poor relationship between mother-in-law and daughter-in-law, (6) lack of social support, (7) unplanned pregnancy and (8) poor living conditions, (9) mother-in-law as the caregiver

*PPD* postpartum depression, *EPDS* Edinburgh postnatal depression scale, *HADS* Hospital Anxiety and Depression Scale, *SDS* self-rating depression scale, *SCL-90* symptom checklist 90, *BDI* beck depression inventory, *CES-D* Center for Epidemiological Studies Depression Scale

### Quantitative synthesis

#### Case-control studies and cohort studies

**Fig. 2:**
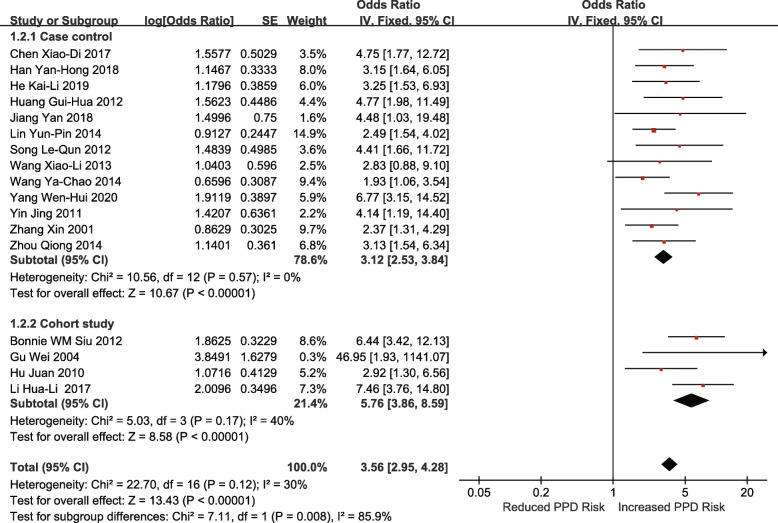
Poor relationship between husband and wife

Seventeen studies (13 case-control studies and 4 retrospective cohort studies) reported that a poor relationship between husband and wife was associated with an increased risk of PPD (OR = 3.56; 95% CI 2.95–4.28; I^2^ = 30%; *p* < 0.00001) (Fig. 2).

**Fig. 3:**
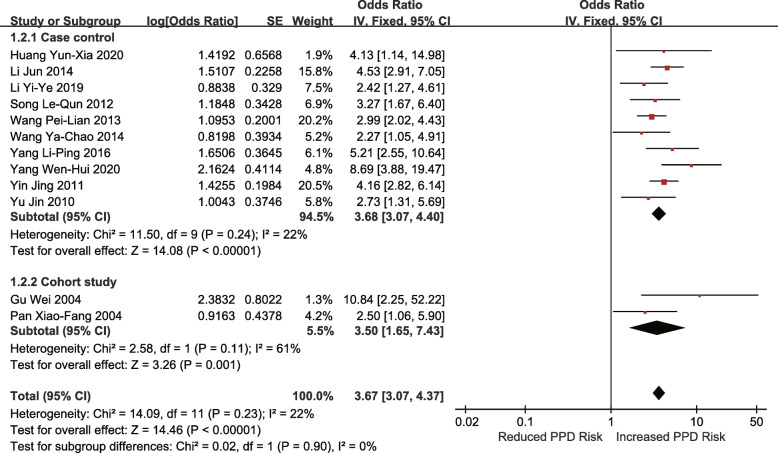
Poor economic foundation

Eleven studies (10 case-control studies, 2 retrospective cohort studies) investigated the association between poor economic foundation and the risk of PPD (OR = 3.67; 95% CI 3.07–4.37; I^2^ = 22%; p < 0.00001) (Fig. 3).

**Fig. 4:**
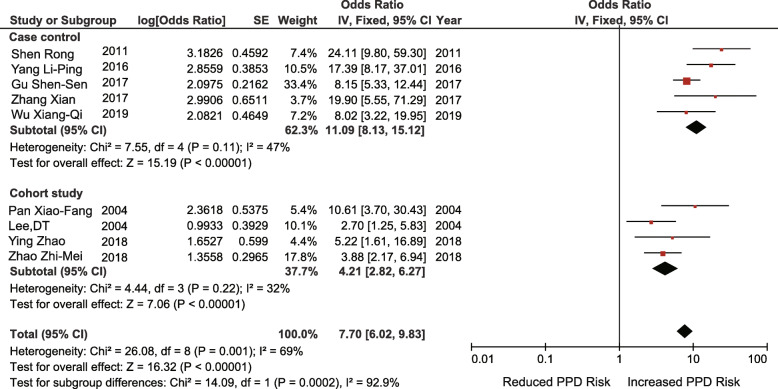
Prenatal depression

Nine studies (5 case-control studies, 4 retrospective cohort studies) investigated the association between prenatal depression and the risk of PPD (OR = 7.70; 95% CI 6.02–9.83; I^2^ = 69%; *p* < 0.00001) (Fig. 4). Thus, poor economic foundation and prenatal depression were significantly related to PPD.

**Fig. 5:**
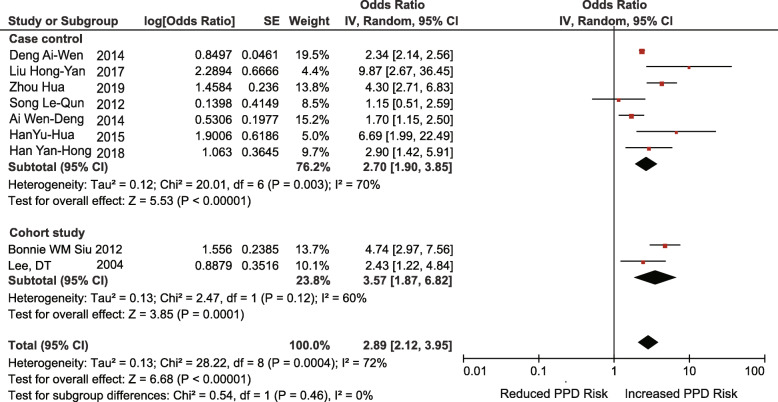
Poor relationship between mother-in-law and daughter-in-law

Nine studies (7 case-control studies, 2 retrospective cohort studies) reported that a poor relationship between mother-in-law and daughter-in-law was associated with an increased risk of PPD (OR = 2.89; 95% CI 2.12–3.95; I^2^ = 72%; *p* < 0.00001) (Fig. 5).

**Fig. 6:**
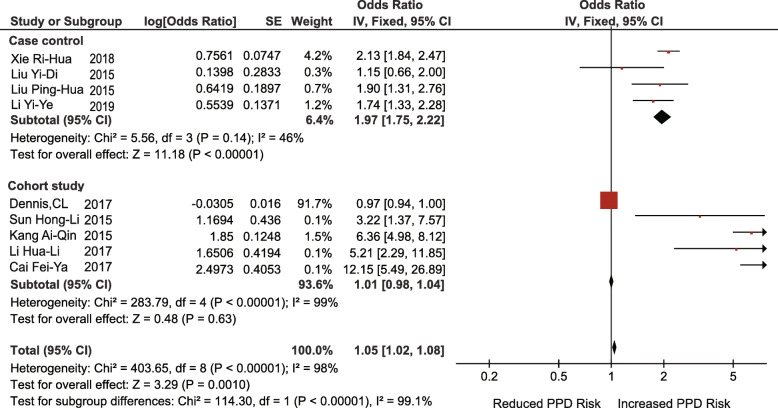
Lack of social support (before adjustment)

**Fig. 7:**
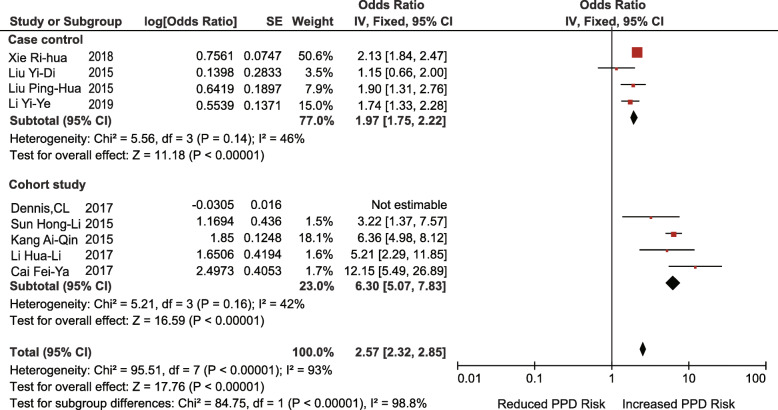
Lack of social support (after adjustment)

Nine studies (4 case-control studies, 5 retrospective cohort studies) investigated the association between a lack of social support and the risk of PPD (OR = 2.66; 95% CI 1.57–4.53; I^2^ = 98%; *p* = 0.0003). After subgroup analysis according to the type of study design, the heterogeneity of the case-control group decreased (I^2^ = 46%, *p* = 0.14), but the heterogeneity of the cohort group was still very high (I^2^ = 99%, *p* < 0.00001). The heterogeneity of the cohort group decreased significantly when one study was excluded [67]. (I^2^ = 42%, *p* = 0.16) (Fig. 6, 7). This may be due to major differences in social support between Chinese-Canadian women and Chinese women [26].

#### Case-control studies

**Fig. 8:**
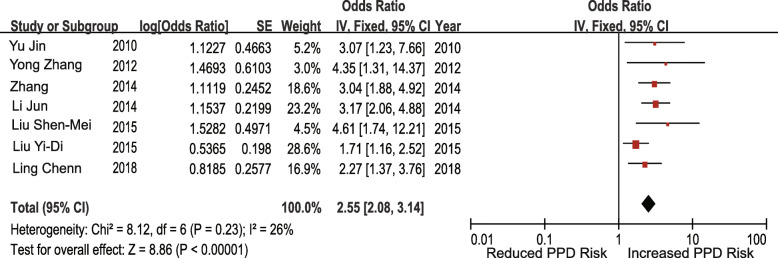
Unplanned pregnancy

**Fig. 9:**
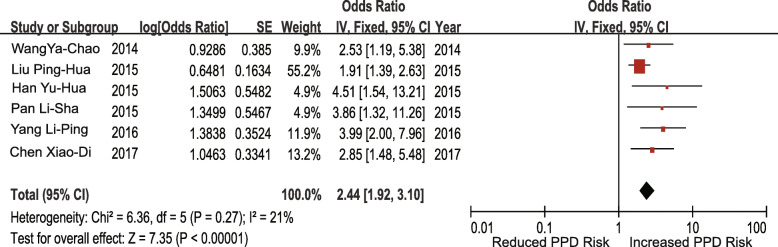
Poor living conditions

**Fig. 10:**
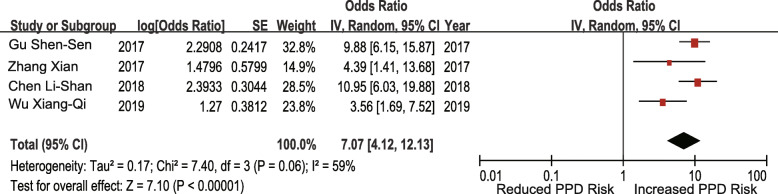
Prenatal anxiety

**Fig. 11:**
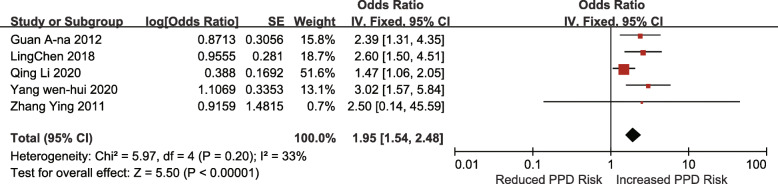
Mother-in-law as the caregiver

Seven studies investigated the association between unplanned pregnancy and the risk of PPD. The pooled OR for unplanned pregnancy was 2.55 (95% CI 2.08–3.14; I^2^ = 26%; *p* < 0.00001) (Fig. 8). Six studies investigated the association between poor living conditions and the risk of PPD. The pooled OR for poor living conditions was 2.44 (95% CI 1.92–3.10; I^2^ = 21%; *p* < 0.00001) (Fig. 9). Four studies investigated the association between prenatal anxiety and the risk of PPD. The pooled OR for prenatal anxiety was 7.07 (95% CI 4.12–12.13; I^2^ = 59%; *p* < 0.00001), and a random-effects model was adopted (Fig. 10). Five studies investigated the association between mothers-in-law as caregivers and the risk of PPD. The pooled OR for mothers-in-law as caregivers was 1.95 (95% CI 1.54–2.48; I^2^ = 33%; *p* < 0.00001) (Fig. 11).

Therefore, unplanned pregnancy, poor living conditions, prenatal anxiety, and mothers-in-law as caregivers were found to be significantly associated with PPD.

#### Heterogeneity test and sensitivity analysis

The results of 51 studies were tested by the Cochrane Q test. The heterogeneity test results of the seven risk factors were low or medium (prenatal depression, poor economic foundation, poor relationship with partner, lack of social support, unplanned pregnancy, mothers-in-law as caregivers, and poor living conditions). However, for the poor mother-in-law relationship and prenatal anxiety, the random-effects model was used because of the high heterogeneity. Among them, the high heterogeneity of the poor relationship between mother-in-law and daughter-in-law may be due to the lack of targeted questionnaire surveys, the setting of the questionnaires being more subjective, and the measurement standard more difficult to unify. The high heterogeneity of prenatal anxiety may be due to the different times of prenatal measurement [17]. In sensitivity analyses, the change model method was used to estimate the point and interval of the OR values of all risk factors to judge the stability of the meta-analysis (Table 2). The point estimates of the combined OR values of the fixed-effects model and the random-effects model were similar, and the interval estimation range of the random-effects model was slightly wider than that of the fixed-effects model. This result indicated that the comprehensive analysis results of the influencing factors in this study were reliable overall.

**Table 2:** Sensitivity analysis of risk factors of PPD

		Fixed effect model		Random effect model	
Risk factors	Type of research	OR	95%CI	OR	95%CI
Prenatal depression	Cohort study	4.21	[2.82, 6.27]	4.40	[2.64, 7.34]
	Case control study	11.09	[8.13, 15.12]	12.72	[7.85, 20.62]
marriage relationship	Cohort study	5.76	[3.86, 8.59]	5.76	[3.27, 10.13]
	Case control study	3.12	[2.53, 3.84]	3.12	[2.53, 3.84]
Mother-in-law relationship	Cohort study	3.84	[2.61, 5.65]	3.57	[1.87, 6.82]
	Case control study	2.37	[2.17, 2.58]	2.70	[1.90, 3.85]
Social support	Cohort study	6.30	[5.07, 7.83]	6.18	[4.10, 9.31]
	Case control study	1.97	[1.75, 2.22]	1.85	[1.52, 2.25]
Economic foundation	Cohort study	3.50	[1.65, 7.43]	3.67	[2.97, 4.55]
	Case control study	3.68	[3.07, 4.40]	4.46	[1.09, 8.20]
Unplanned pregnancy	Case control study	2.55	[2.08, 3.14]	2.64	[2.04, 3.40]
Prenatal anxiety	Case control study	7.93	[5.76, 10.90]	7.07	[4.12, 12.13]
Mother-in-law as the caregiver	Case control study	1.95	[1.54, 2.48]	2.11	[1.52, 2.93]
Living conditions	Case control study	2.44	[1.92, 3.10]	2.65	[1.96, 3.57]

*ORs* odds ratios, *CIs* corresponding 95% confidence intervals

#### Publication bias

**Fig. 12:**
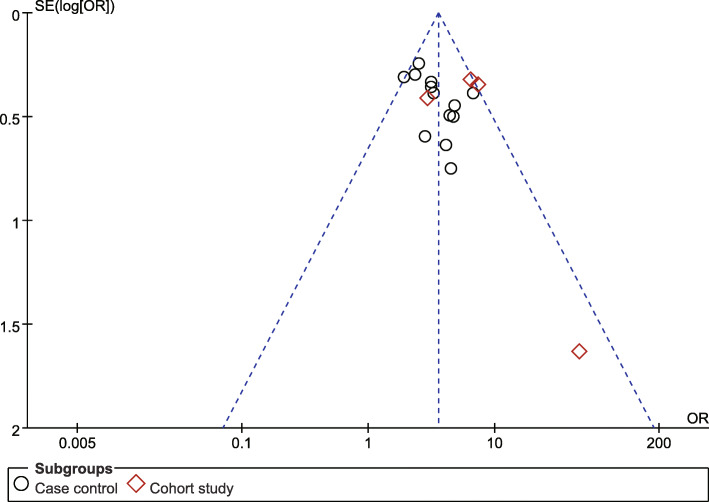
Poor relationship between husband and wife funnel plot

**Fig. 13:**
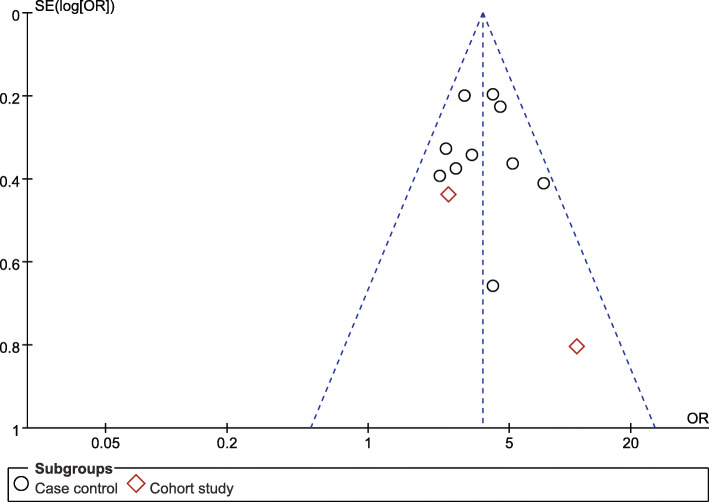
Poor economic foundation funnel plot

Publication bias was evaluated via the visual analysis of funnel plots. The funnel plot generally appeared to be symmetrical, indicating no publication bias (Fig. 12, 13).

## Discussion

PPD is a crucial part of the spectrum of mood disturbances affecting postpartum women. A variety of factors affect the physical and mental health of pregnant women. Thus, identifying alterable risk factors for PPD and controlling them at an early stage are essential for the treatment and prevention of this condition.

The psychosocial risk factors for PPD in Chinese women identified in this meta-analysis mainly included three kinds: prenatal emotional factors (prenatal anxiety and prenatal depression), social demographic factors (poor marital relationship, poor living conditions, lack of social support, and unplanned pregnancy) and social and interpersonal factors (poor relationship between husband and wife, poor relationship between mother-in-law and daughter-in-law,and mother-in-law as the caregiver). First, prenatal anxiety and depression were significantly associated with an increased risk of PPD, as confirmed by some Western studies. An Italian study [68] showed that women with depression or anxiety during pregnancy and a lack of support from family and friends were at a higher risk of PPD. This result has also been confirmed in China. According to the report of Lee [69], most PPD was the continuation of prenatal psychological problems and emotional disorders, indicating a significant correlation between prenatal psychological status and the occurrence of PPD.

Another explanation for the effect of prenatal emotional distress is physiological changes. For example, excessive anxiety and depression in pregnant women may lead to a series of physiological and pathological reactions, such as a decrease in norepinephrine secretion and changes in other endocrine hormones, which may lead to the weakening of uterine contractions, a prolonged stage of labor, and increased bleeding. These challenges further aggravate the anxiety of pregnant women and lead to an increased risk of developing PPD [70].

Second, this study found that social demographic factors were also risk factors for PPD, such as a poor economic foundation, poor living conditions, a lack of social support, and unplanned pregnancy. Among them, the economic foundation of the family had an important effect on the psychological status of the mother. Previous reviews suggested that the status of the family’s economic income was positively related to the level of stress in pregnant women. Yu [46] suggested that after adjustments were made for other related factors, the incidence of PPD among women who were worried about family economic status was 3.162 times higher than among those who did not worry about it. The probable explanation may be that after childbirth, the cost of raising the baby and the basic cost of living for the family significantly increases. If the family income is insufficient, it will lead to high levels of pressure for pregnant women and can easily cause negative emotions. In recent years, with China’s two-child policy, raising multiple children in a family increases the family’s financial burden, which may be a factor of PPD. A study in Turkey shows that there was a significant relationship between monthly income and depression, which was similar to the results of the present study [71].

In addition, previous studies have shown that social support was a protective factor against PPD, and as far as mothers were concerned, the greatest social support comes from their husbands. Xiong et al. [72] suggested that puerperae with spousal support were much less likely to develop PPD. A Chinese study also confirmed that high levels of social support can reduce the risk of PPD, with other factors were fixed [27]. Our findings are generally consistent with those of previous reviews. Therefore, giving adequate social support to parturients during the puerperal period can help them get through this critical period smoothly.

Third, in this study, the interpersonal risk factors for PPD were a poor relationship between husband and wife, a poor relationship between mother-in-law and daughter-in-law, and mother-in-law as the caregiver. The poor relationship between husbands and wives, as an important factor affecting human physical and mental health, has attracted the close attention of researchers worldwide. Zhang [73] suggested that the quality of the husband-wife relationship was mainly reflected in the quality of the husband’s care for his wife, and women who were less satisfied with their husband’s care were more likely to have depression. Poor marriage and family relationships will not only reduce maternal social support but also become a maternal stressful life event, which brings about an increased risk of developing PPD. This study was confirmed in a Polish study. Malus et al. [74] confirmed the significance of the marital relationship in the development of PPD. A sense of closeness and intimacy in the relationship were associated with better mood and a greater ability to cope with the difficulties of labor, puerperium, and caring for a newborn baby.

In addition, the results of this study found that the risk factors for PPD related to Chinese cultural characteristics included the mother-in-law as the caregiver and a poor relationship between mother-in-law and daughter-in-law.

From the perspective of family structure, Chinese families have a close relationship, intergenerational support shows a circular mode, and it is very common to expand families, especially families with three generations living together. Even if the whole family emigrates to other countries, it is still common for parents to live together with their children’s families. The kinship network linked by blood relationship is complicated, and the conflict increases accordingly [75].

In Western countries, nuclear families are the main organizational form; they attach importance to the development of personality, pay attention to independence and privacy, and think that the lifestyle of several generations living together seriously infringes upon the privacy of individuals, which is unbearable. Therefore, adult children live independently of their parents. Kinship is more distant than that of China, and contradictions and intergenerational conflicts are correspondingly less [76].

Traditionally, mothers-in-law exercise significant power in the family and are a major influence on the postpartum care of new mothers. In China, due to the influence of doing-the-month culture, mothers and newborns are mostly cared for by their mothers-in-law. The strain between mothers-in-law and daughters-in-law is a sensitive problem and may be a cause of PPD in China. Steinberg [77] indicated that the strain between mothers-in-law and daughters-in-law often offset the benefits of assistance and may even contribute to negative mood during the postpartum period. In traditional Confucian philosophy, the new mother should be considered a good daughter-in-law if she behaves in a way that is respectful at home and is obedient to her in-laws and husband [78]. The relationship between women and their parents-in-law is based on the environment rather than consanguinity. Sometimes they are reluctant to express their own feelings and opinions to their in-laws. New mothers feel very stressed when they have opinions different from their care providers. The situation may become even worse when conflicts occur with mothers-in-law. Because of the differences in backgrounds, values, identity, and logic of ideas, conflicts with respect to childcare between women and their mothers-in-law become prominent [18].

### Limitations

This study had several inevitable limitations. First, some risk factors have received less attention; for example, non-uniform measurement standards and statistical difficulties have not been combined, such as the type of residence, postpartum wound recovery, postpartum work stress, and maternal occupation. Second, PPD is the result of the interaction of multiple factors, but due to methodological limitations, it is difficult to investigate the interaction among risk factors. Third, in terms of language selection, this study only includes literature in Chinese and English, which may lead to bias in the comprehensiveness of the literature search, thus affecting the research results and the intensity of the argument.

In addition, this study covers a wide range of research sites, including pregnant Chinese women in mainland China and Hong Kong, and Canadian immigrants. Although some Chinese individuals have emigrated to other countries, they have been influenced by traditional Chinese culture for a long time; thus their personality and their family structure are still similar to those of individuals in their motherland. In particular, many Chinese immigrant women continue to observe the Chinese traditional postpartum practices.

## Conclusion

In conclusion, psychosocial risk factors for PPD mainly include prenatal depression, prenatal anxiety, a poor economic foundation, a poor relationship between husband and wife, a poor relationship between mother-in-law and daughter-in-law, a lack of social support, unplanned pregnancy, the mother-in-law as the caregiver, and poor living conditions. These psychosocial risk factors are meaningful for identifying mothers “at-risk” during pregnancy even earlier. Meanwhile, some psychosocial interventions targeting these risk factors may be conducted during the pregnancy period to prevent PPD, such as interpersonal psychotherapy, mindfulness therapy, and psychoeducational programs.

## Data Availability

Data will be available from the corresponding author upon reasonable request.

## Abbreviations

PPD: Postpartum depression
EPDS: Edinburgh Postnatal Depression Scale
HADS: Hospital Anxiety and Depression Scale
SDS: Self-rating depression scale
SCL-90: Symptom checklist 90
BDI: Beck depression inventory
CES-D: Center for Epidemiological Studies Depression Scale
VIP: the China Science and Technology Journal Database
CNKI: China National Knowledge Infrastructure
CBM: China Biology Medicine Disc
RRs: Relative risks
ORs: Odds ratios
CIs: Corresponding 95% confidence intervals
NOS: Newcastle-Ottawa Scale
